# Interactions between Phosphatidylcholine and Kaempferol or Myristicin: Langmuir Monolayers and Microelectrophoretic Studies

**DOI:** 10.3390/ijms22094729

**Published:** 2021-04-29

**Authors:** Paulina Laszuk, Aneta D. Petelska

**Affiliations:** Bioelectrochemistry Laboratory, Faculty of Chemistry, University of Bialystok, Ciolkowskiego 1K, 15-245 Bialystok, Poland; p.laszuk@uwb.edu.pl

**Keywords:** kaempferol, myricetin, phosphatidylcholine, Langmuir monolayer, Brewster angle microscopy, complex formation equilibria

## Abstract

Flavonoid compounds are known for their antibacterial, anti-inflammatory, and anticancer properties. Therefore, they can influence membrane properties that interest us, modifying both their structure and functions. We used kaempferol (K) and myricetin (M) as representatives of this group. We investigated the influence of the abovementioned compounds on model cell membranes’ properties (i.e., Langmuir monolayers and liposomes). The basic research methods used in these studies were the Langmuir method with Brewster angle microscopy and microelectrophoresis. The π–A isotherms were registered for the pure components and mixtures of these compounds with phosphatidylcholine (PC) in appropriate volume ratios. Using mathematical equations, we established that kaempferol, myricetin, and the lipids formed complexes at 1:1 ratios. We derived the parameters characterizing the formed complexes, i.e., the surfaces occupied by the complexes and the stability constants of the formed complexes. Using the microelectrophoretic method, we determined the dependence of the lipid membranes’ surface charge density as a function of the pH (in the range of 2 to 10) of the electrolyte solution. The presented results indicate that the PC membrane’s modification with kaempferol or myricetin affected changes in the surface charge density and isoelectric point values.

## 1. Introduction

Langmuir monolayers are monomolecular, insoluble films formed at the water/air interface. The monomolecular layers show an amphiphilic structure: the surfactant’s polar part is immersed in water, while the hydrocarbon chain is directed towards the air [1,2]. The simplest and most commonly used models of cell membranes are monolayers. The created model allowed, among other things, the analysis of the interactions of compounds on membranes and inside the cell [3]. It allowed for elucidating, in detail, the mechanism of transport of medicinal substances. Important information on the properties of the monolayer, such as the packing of molecules at the water–air interface, is provided by the graph of the surface pressure (π) versus the surface area occupied by a single molecule in the monolayer (A) (π–A isotherm) [4].

The second natural membrane models were liposomes used in medicine and many other science branches because of their construction. Liposomes can be formed from natural or synthetic lipids. Regardless of whether they consist of natural or synthetic lipids, liposomes are biocompatible and biodegradable, making them suitable for biophysical and medical research.

Compounds of natural origin have been used in medicine and herbalism for a long time. However, in recent years, the interest in natural-origin substances as a source of biologically active compounds has returned. These substances are known for their antibacterial, anti-inflammatory, and anticancer properties. Therefore, they can influence the membranes’ properties that interest us, modifying both their structure and functions. Such properties are characterized by polyphenol compounds, which in plants are dyes, antioxidants, and have protective functions. Provided to the body by food, they show high biological activity and positively affect the circulatory system’s work.

Flavonoids are the most abundant group of polyphenolic compounds found in nature. Although over 4000 flavonoids are known [5], only a few, such as quercetin, kaempferol, myricetin, apigenin, and luteolin, have been described as important components of the human diet. Flavonoids in plants perform protective functions against reactive oxygen species (ROS), which are formed in the process of photosynthesis [6]. Flavonoids can prevent oxidative damage to living organisms caused by ROS and/or reactive forms of nitrogen (RNS) [7].

**Figure 1:**
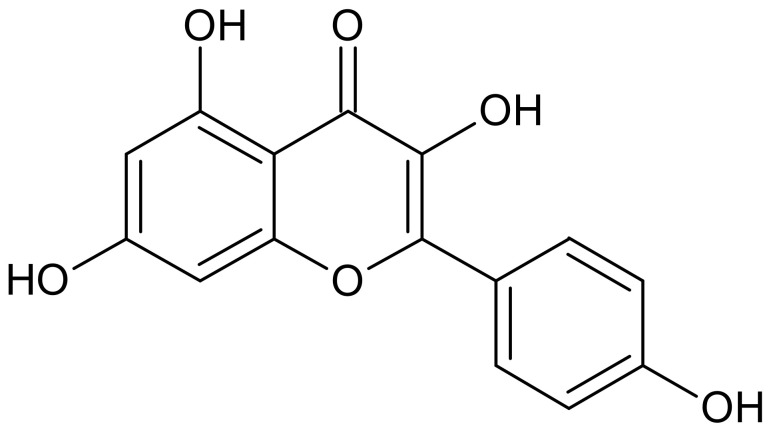
Structure of kaempferol.

**Figure 2:**
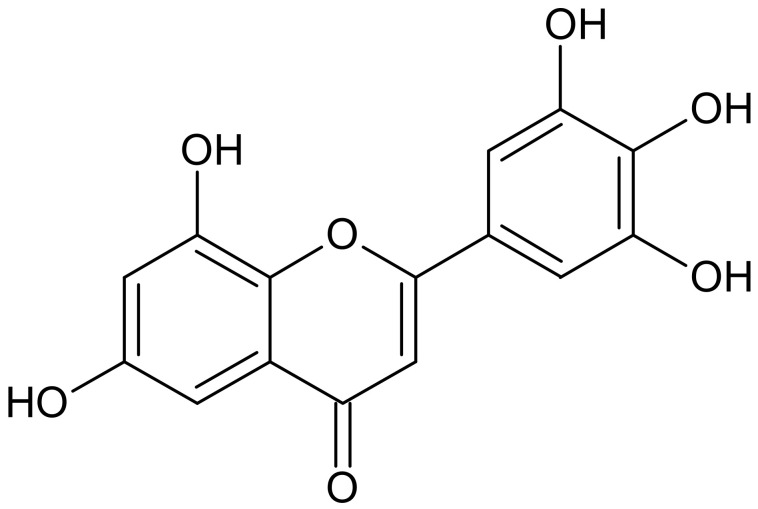
Structure of myricetin.

The characteristic feature of each flavonoid molecule is a skeleton composed of 15 carbon atoms that contains two aromatic rings, linked together by a three-carbon bridge that is part of a heterocyclic, pyran ring (Figure 1 and Figure 2) [8]. Flavonoids may also exist as glycosides, which, in contrast to aglycones, possess bound carbohydrate groups or contain methyl groups. The hydroxyl groups of flavonoids give these compounds the properties of weak acids that can deprotonate to form anionic species, depending on their pKa and the pH of the local environment [8].

Flavonoids play an important role in the bioavailability of trace amounts of metal ions in the human body. They can also be used in detoxification with heavy metals, such as lead, for example. This is due to the ability of the flavonoid hydroxyl groups to chelate free redox metal ions and form stable complexes, which are then removed from the body [9]. The antioxidant/pro-oxidative properties of flavonoids are mainly related to the number and position of hydroxyl groups and their ability to chelate redox metals (Cu, Fe). Jamowa et al. [8] studied structurally different flavonoids, such as myricetin, 3′, 4′- dihydroxy-flavone, taxifolin, and 4-hydroxycoumarin, alone or as interacting with Cu^2+^ ions. The results confirm the efficient binding of cupric ions with flavonoids [8].

The representatives of the flavonoids group which we used in our study include kaempferol and myricetin. Kaempferol (Figure 1) is a yellow solid that dissolves well in organic solvents. It has anticancer, antioxidant, and anti-inflammatory properties [10,11]. It is also called the guardian of a healthy heart, because it increases the elasticity of blood vessels. Kaempferol is abundant in tea, broccoli, apples, strawberries, and beans [12]. Kaempferol was experimentally and theoretically investigated for its ability to scavenge potentially highly damaging hydroxyl and superoxide anion radicals [13].

Myricetin (Figure 2) is a popular plant-derived flavonoid and is well known for its nutritional value. It is one of the key ingredients in various dishes and drinks. The compound exhibits a wide range of effects including strong antioxidant, anticancer, antidiabetic, and anti-inflammatory properties [14,15,16,17]. One of its beneficial biological effects is its neuroprotective activity, showing preclinical activities against Alzheimer’s, Parkinson’s, and Huntington’s diseases, and even in amyotrophic lateral sclerosis [18]. Myricetin is a yellow organic compound found in walnuts, red wine, and black currants [19]. Myricetin is the most potent inhibitor among flavanols. The results obtained for myricetin confirm the relationship between its structure and function, and the number and position of the OH group on the ring [20]. Myricetin has also been identified as the most potent inhibitor (of the 16 important flavonoids) of mammalian DNA polymerases and human topoisomerases in in vitro studies [21].

To our knowledge, there are few data on interactions between the monolayer and liposomes and flavonoids (e.g., kaempferol and myricetin). In particular, there are no data on the physicochemical and electrical properties of flavonoid (kaempferol or myricetin)-modified monolayer and bilayer lipid membranes. Therefore, this work aimed to analyze the influence of these flavonoids on the surface pressure, the surface area occupied by molecules, and the surface charge density of model cell membranes (monolayers and liposomes). These parameters are very important in characterizing both natural and artificial lipid membranes. Previously, we have described changes in the surface pressure, the area occupied by the particles, and surface charge density, which affects, for example, the composition of membrane phosphatidylcholine-diosgenin [22,23] and phosphatidylcholine-lipoic acid [24].

Particularly important for understanding the phenomena occurring on the surface of the membrane is their quantification, which is necessary for a full interpretation. We conduct it here, using simple mathematical relationships to theoretical models relating to the quantitative description of the phenomena occurring between the membrane’s elements and between them and the environment.

Then, through theoretical descriptions of complex formations, we can verify the experimental and theoretical data. This allows for quantifying parameters describing the equilibrium, i.e., surface pressure, surface charge density, stability constants, and complex formation energies. As there are no precise data on the stability constants for connection formation in the literature, our team is the first to define these parameters and present them. In our opinion, this is an original contribution to the knowledge on the biochemistry and biophysics of lipid membranes.

The research presented in this article is a continuation of our research on the interaction of model membranes (phosphatidylcholine) and flavonoid molecules (kaempferol and myricetin). In these systems, the formation of the complex was assumed in a ratio of 1:1. We also show a comparison of the parameters of the complexes (stability constants and surface area). We also analyze the effect of the pH of the electrolyte solution and the membrane’s composition on the surface charge to describe better the interactions in phosphatidylcholine (PC) membranes modified with selected flavonoids (kaempferol and myricetin). As these parameters influence interactions between membranes and biologically active compounds, we believe that the data presented below may help us to understand membrane-binding mechanisms. There has been a growing interest in research into therapeutic research and therapeutically effective research in recent years. It turns out that such indicators are flavonoids. They show promise in the treatment of a variety of diseases and, at the same time, have their natural source in many plants. Information on the interactions between selected flavonoids (e.g., kaempferol and myricetin) and components of natural biological membranes may contribute to the development of medicine and a better understanding of the processes taking place in biological membranes.

## 2. Results and Discussion

The influence of kaempferol and myricetin on biological membranes’ physicochemical and electrical properties is not fully understood but is very important. Flavonoids exert multiple positive effects at the level of cells and the body as a whole including the antitumor effect. They have properties that regulate some neoplastic mechanisms, e.g., reduce the expression of some oncogenes, inhibit uncontrolled cell division, and induce processes leading to cancer cells’ death. Nevertheless, in the literature, there are few data on the effect of flavonoids on the physicochemical and electrical properties of model systems, where the lack of quantitative descriptions of equilibria is noticeable.

### 2.1. Monolayer Experiment

**Figure 3:**
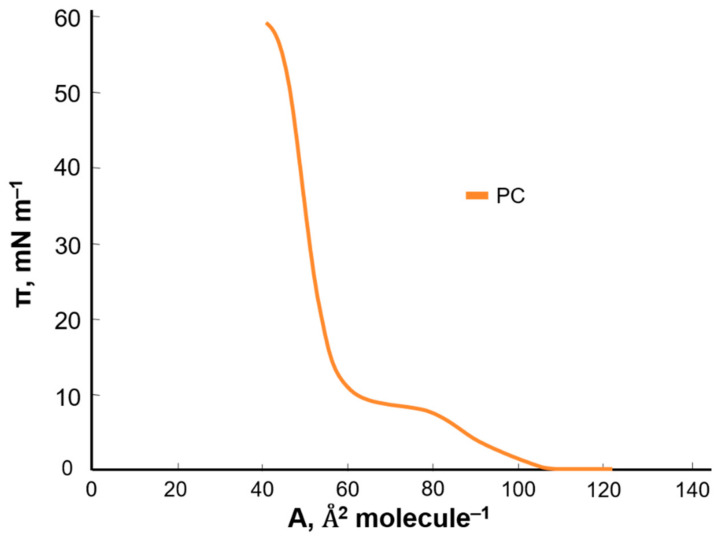
Surface pressure-area (π–A) isotherms of phosphatidylcholine.

**Figure 4:**
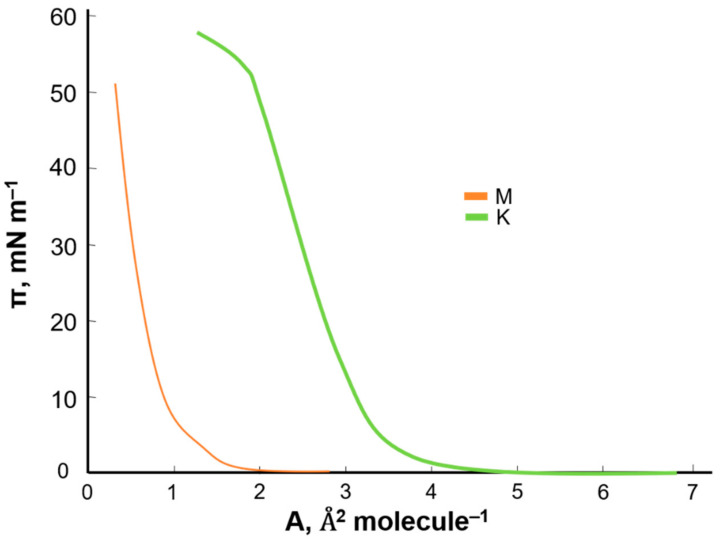
Surface pressure-area (π–A) isotherms of myricetin (M) and kaempferol (K).

To examine the effect of kaempferol and myricetin on the basic components of cell membranes (i.e., phosphatidylcholine), the π–A isotherms of pure substances were prepared. Using the Langmuir method with Brewster angle microscopy, π–A isotherms were recorded for pure compounds: PC (Figure 3), kaempferol, and myricetin (Figure 4).

Based on the obtained curves, it can be concluded that, with the simultaneous increase in the surface pressure, the surface area per single molecule decreased. The π–A isotherm shows the basic phase states: gas (G), extended liquid (LE), and condensed (LC) with the simultaneous occurrence of intermediate states (G/LE, LE/LC). At the maximum pressure value (collapsing pressure), the monolayer collapsed. The specific surface area of the PC was 59 Å^2^ molecule^−1^. In the case of kaempferol and myricetin, intermediate states were not included in the graph. The two compounds tested had a much lower specific surface area than PC. The average area determined for a single molecule in a monolayer for kaempferol was 4 Å^2^ molecule^−1^ and for myricetin 1 Å^2^ molecule^−1^.

**Figure 5:**
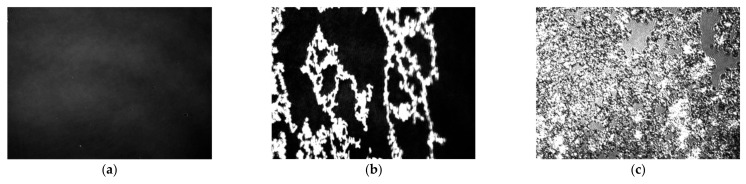
BAM images taken for kaempferol at three surface pressures: (**a**) 0.08 mN/m, (**b**) 30.13 mN/m, and (**c**) 54.47 mN/m.The images were captured during compression at constant temperature and surface pressure in the field of view 3.6 × 4.0 mm. A black glass plate immersed in the subphase absorbed the refracted beam. The resolution of image was approx. 6 μm/pixel.

**Figure 6:**
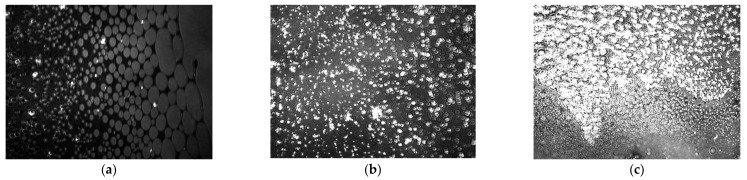
BAM images were taken for myricetin for three surface pressure: (**a**) 0.21 mN/m, (**b**) 20.61 mN/m, (**c**) 48.93 mN/m.The images were captured during compression at constant temperature and surface pressure in the field of view 3.6 × 4.0 mm. A black glass plate immersed in the subphase absorbed the refracted beam. The resolution of image was approx. 6 μm/pixel.

The registration of π–A isotherms with BAM’s simultaneous use enabled the visualization of phase transitions, providing more detailed information on the processes taking place [25,26]. BAM images obtained for kaempferol (Figure 5) and myricetin (Figure 6) are shown below.

Due to the same phases during the film compression for both tested compounds, the description of the photos for Figure 5 and Figure 6 are presented. In the first picture for both myricetin and kaempferol, we observed the gas phase (G) (Figure 5a or Figure 6a) where the molecules’ interaction was limited due to the distance between them. The molecules of both compounds contained polar -OH groups. Some of the molecules may lie flat on the surface of the subphase, while others may be perpendicular to its surface. The hydrocarbon chains, being the non-polar part, may be bent in several places.

Then, we observed the transition to the liquid phase (LE/LC) (Figure 5b or Figure 6b), which is caused by the gradual organizing of amphiphilic molecules. Polar heads begin to interact more with each other. On the other hand, hydrophobic chains are directed towards the gas phase—they are stiffened. The molecules that make up the monolayer behave similarly to a two-dimensional isotropic liquid.

Subsequent compression results in tighter packing and more intermolecular interactions. The decreasing distance between the polar heads allows for stronger interactions between them, for example, the formation of hydrogen bonds. The next images show the coexistence of a 2D monolayer (dark areas) and 3D crystals under collapse pressure—the solid phase (S) (Figure 5c or Figure 6c); this proves the perpendicular orientation of the molecules with the water’s surface. After exceeding the collapse pressure, we see an image practically filled with 3D structures. Due to the high flexibility of the monolayer, several-layered crystallites with an irregular surface were observed [22,27,28].

### 2.2. Mixed Monolayer Experiment and Theoretical Consideration

In two-component monolayers (lipid (L)–flavonoid (F) (i.e., kaempferol or myricetin)), both substances tend to form complexes at a 1:1 ratio (Equation (1)). According to data in the literature, this is dictated by the system’s aspiration to achieve equilibrium and, thus, obtain the largest value of the stability constant [29].

Formation of a complex in a 1:1 ratio may be represented by the following equation:

(1) L+F⇔L−F

The equilibrium state of the complex (1:1) is described by the system of equations:

(2) $$a_{L}S_{L}+a_{F}S_{F}+a_{L-F}S_{L-F}=1$$

(3) $$a_{L}+a_{L-F}=c_{L}$$

(4) $$a_{F}+a_{L-F}=c_{F}$$

(5) $$K_{L-F}a_{L}a_{F}=a_{L-F}$$

(6) $$x_{F}\left(a_{F}+a_{L}\right)=a_{F}$$

where *a_L_*, *a_F_*, and *a_L_*_–*F*_ (mol m^−2^) are the surface concentrations of components L and F; *c_L_* and *c_F_* (mol m^−2^) are the total surface concentrations of components L and F; *S_L_*, *S_F_*, and *S_L_*_–*F*_ (m^2^ mol^−1^) are the surface areas occupied by 1 mol of components L and F and complex L–F; *K_L_*_–*F*_ (m^2^ mol^−1^) is the stability constant of complex L–F, and *x_F_* are the mole fractions of component F.

The full theoretical part concerning the mathematical equations describing the complex formation process, Equations (2)–(6) and the formulas enabling the calculation of the stability constants Equation (7), the specific areas of the created complexes, Equation (8) and the energy of complex formation was presented in a previous paper [30].

Solving a system of equations ultimately yields the following equations [24,30]:

The equation describing the stability constant of the complex:

(7) $$K_{L-F}=\frac{S_{F}^{3}C_{F\left(x_{F}=1\right)}^{\prime }-2S_{L}S_{F}-S_{L}^{3}c_{L\left(x_{F}=0\right)}^{\prime }}{S_{F}-S_{L}+S_{L}^{2}c_{L\left(x_{F}=0\right)}^{\prime }+S_{F}^{2}c_{F\left(x_{F}=1\right)}^{\prime }}$$

The equation describing the area occupied by one molecule of the complex:

(8) $$S_{L-F}=\frac{\left(S_{L}S_{F}+c_{L(x_{F=0)}}^{\prime }c_{F\left(x_{F=1}\right)}^{\prime }S_{L}^{2}S_{F}^{2}\right)\left(S_{L}+S_{F}\right)}{S_{L}^{3}c_{L\left(x_{F=0}\right)}^{\prime }+S_{F}^{3}c_{F\left(x_{F=1}\right)}^{\prime }}$$

To determine the quantities described by Equations (7) and (8) for the experimental data, the slopes of the tangents in the ranges, including x_F_ → 0.00 and x_F_ → 1.00, should have been included in the above equations.

To validate the results of the stability constant and the area occupied by a molecule of the complex, the slopes of the tangents at the points x_F_ → 0.00 and x_F_ → 1.00 should be determined using the 9 and 10 equations [24,30]:

(9) $$C_{L\left(x_{F}=0\right)}^{\prime }=\frac{K_{L-F}\left(S_{L}-S_{L-F}\right)-S_{L}S_{F}}{S_{L}^{2}\left(S_{L}+K_{L-F}\right)}$$

(10) $$C_{F\left(x_{F}=1\right)}^{\prime }=\frac{-K_{L-F}\left(S_{F}-S_{L-F}\right)-S_{L}S_{F}}{S_{F}^{2}\left(K_{L-F}-S_{F}\right)}$$

Using these formulas enabled us to compare the slopes of the curves obtained from the experimental data and calculated using the theoretical equations.

The lipid (PC)–F complex formation energy was calculated using Equation (11):

(11) $$-logK=\frac{∆G^{{}^{\circ}}}{2.3RT}$$

where *K* (m^2^ mol^−1^) is the stability constant of the lipid–F complex, Δ*G°* (J mol^−1^) is the lipid–F complex formation energy, *R* (J mol^−1^ K^−1^) is the gas constant, and *T* (K) is the temperature.

#### 2.2.1. Phosphatidylcholine–Kaempferol Complex

**Figure 7:**
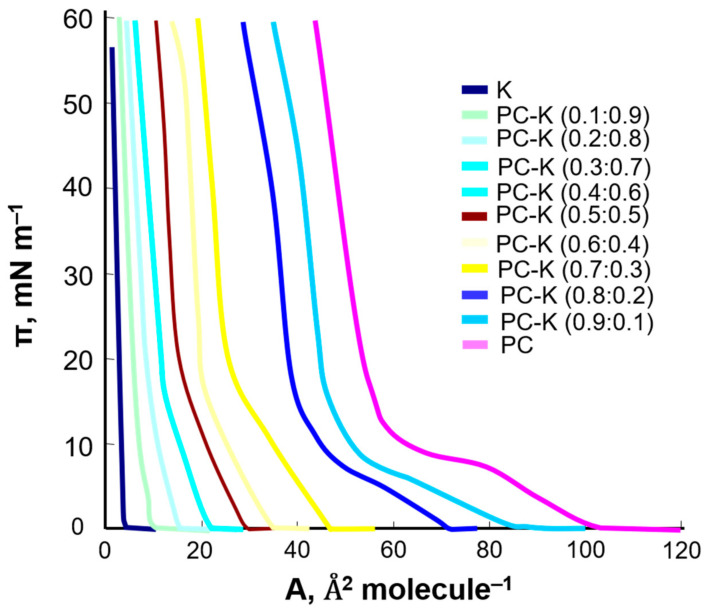
Surface pressure–area (π–A) isotherms of mixed PC–K monolayer.

Phosphatidylcholine was modified with the flavonoids kaempferol and myricetin. A series of solutions with various concentrations of the tested compounds were prepared, and the π–A isotherms were registered (Figure 7).

The obtained curves showed that the surface area occupied by a single molecule in the monolayer decreased with the increase in the kaempferol content in the PC–K mixture. The 1:1 PC–K complex was assumed to exist in monolayers composed of phosphatidylcholine and kaempferol Equations (1)–(3). It was characterized by the stability constant, K_PC–K_ (Equation (4)).

**Figure 8:**
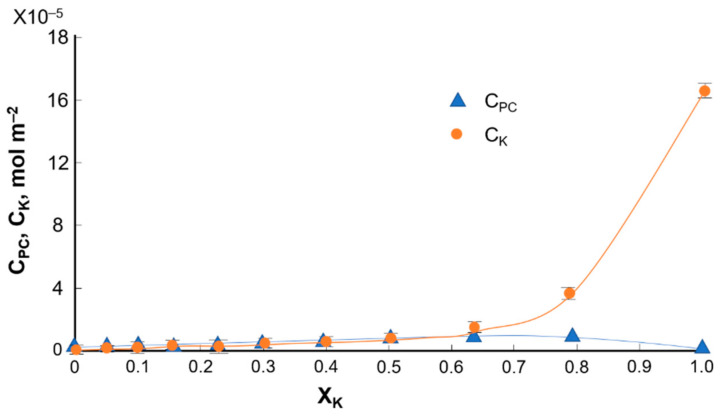
Dependence of the total surface concentration of PC (C_PC)_ and K (C_K_)) vs. the mole fraction of K.

To determine the stability constant of the K_PC–K_ complex and the S_PC–K_-specific surface, the relationship between the surface concentrations of phosphatidylcholine and kaempferol was plotted as a function of the molar fraction of kaempferol; the experimental values are indicated by the points and the theoretical values by the curves (Figure 8).

The area per PC–K complex, S_PC–K_ = 64 Å^2^ molecule^−1^, and the stability constant, K_PC–K_ = 2.8 × 10^6^ were calculated by inserting the experimental data into Equations (7) and (8). Using the values calculated for S_PC-K_ and K_PC-K_ in Equations (9) and (10), theoretical c’_PC_ and c’_K_ values were calculated and compared with the slopes of lines tangent to the experimental data at points x_K_ = 0 and x_K_ = 1.

The S_PC–K_ value obtained this way was higher than the area of a PC molecule, S_PC_ = (52 Å^2^ molecule^−1^), but slightly lower than the sum of the areas of phosphatidylcholine and kaempferol (SPC + SK = 63 Å^2^ molecule^–1^).

Additionally, the value of the complex formation energy (Gibbs free energy) [16] was calculated according to the Equation (11) and equal to −37 kJ mol^−1^.

#### 2.2.2. Phosphatidylcholine–Myricetin Complex

**Figure 9:**
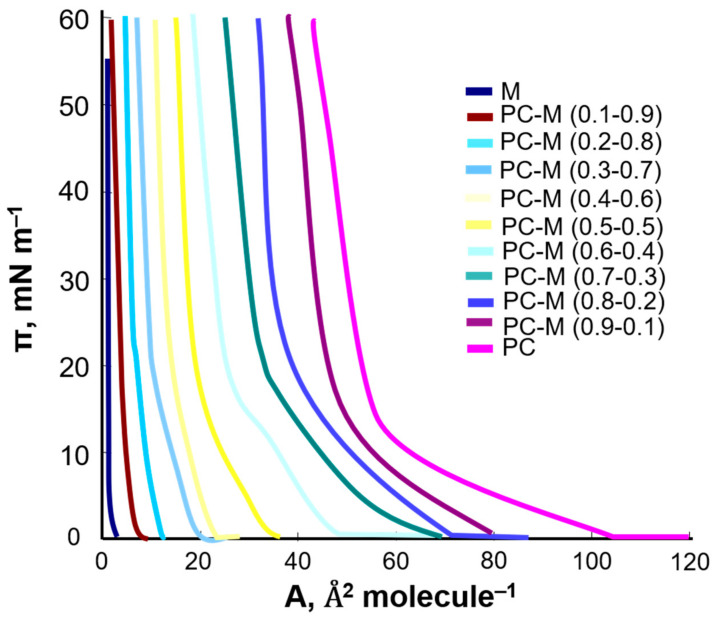
Surface pressure–area (π–A) isotherms of mixed PC–M monolayer.

In order to test the possibility of the formation of the 1:1 complex in the phosphatidylcholine–myricetin system, π–A isotherms were registered for mixed monolayers (Figure 9).

**Figure 10:**
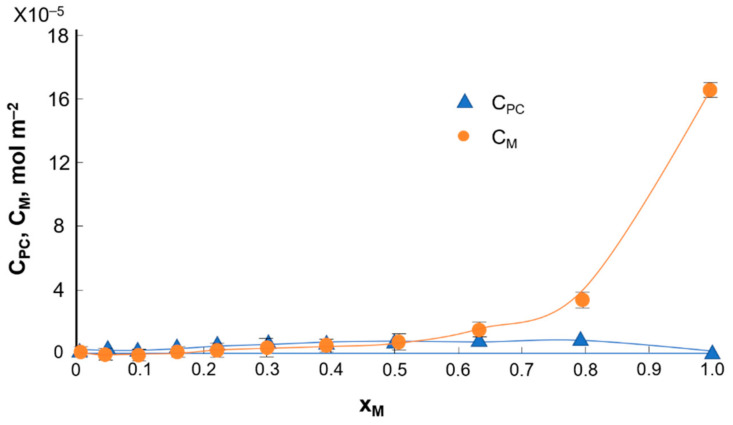
Dependence of the total surface concentration of PC (C_PC)_ and M (C_M_) vs. the mole fraction of M.

The above curves show that the addition of myricetin reduces the surface area occupied by a single molecule in the monolayer for the PC–M system This made it possible to plot the dependence of the total surface concentration of PC (C_PC_) and M (C_M_) vs. the mole fraction of M (Figure 10).

Taking into account the slope of the curves, K_PC–M_ = 2.1 × 10^6^ m^2^ mol^−1^ and S_PC–M_ = 60 Å^2^ molecule^−1^ were calculated (Equations (7) and (8)). Additionally, the experimental value of the specific surface area of the complex was compared with the theoretical value (S_PC_ + S_M_ = 59 Å^2^ molecule^−1^ + 1 Å^2^ molecule^−1^ = 60 Å^2^ molecule^–1^). Both data assumed the same value, which confirms the theoretical considerations about the formation of the 1:1 complex between the components.

Additionally, the value of the complex formation energy (Gibbs free energy) [16] was calculated according to the Equation (11) and equal to −36 kJ mol^−1^.

The data obtained for both PC-K and PC–M complexes are summarized in the table below (Table 1).

The theoretical data, denoted by lines, and the experimental data, denoted by points, are illustrated in Figure 8 and Figure 10. Considering the good agreement between the theoretical and experimental values, it can be concluded that a 1:1 PC–flavonoid complex was formed in the monolayer. During our investigations, we assumed the formation of a PC–flavonoid complex in the monolayer. As flavonoids may also exist as glycosides, the hydroxyl groups of flavonoids can be deprotonated to form anionic species. Therefore, between phosphatidylcholine and flavonoids can form complexes that arise by producing a connection between the −^(+)^N(CH_3_)_3_ group from the phosphatidylcholine molecule and −O^(−)^ group of flavonoids. Therefore, the obtained results can be compared with the previously presented data on phosphatidylcholine–diogenin complexes.

The stability constant of the PC–flavonoid was approximately 2.5 × 10^6^ m^2^ mol^−1^, whereas the stability constant of the PC–diosgenin complex was 6.5 × 10^5^ m^2^ mol^−1^ [31]. These values are relatively high and allow one to argue that the discussed systems have a similar structure. The relatively high stability of PC–flavonoid provides additional evidence for the prevalence of the 1:1 complex in mixed phospholipid–flavonoid monolayers.

Knowledge of this thermodynamic function provides information concerning the nature and type of bonding in the tested systems and groups taking part in the complex-forming reactions, for which there are many areas of application in chemistry, biology, and medicine. The complex formation energy value for the PC–kaempferol and PC–myricetin systems was approximately −36 kJ mol^−1^. The values presented above are close to those determined earlier for similar systems (e.g., PC–diosgenin systems were −33 kJ mol^−1^) [31].

**Figure 11:**
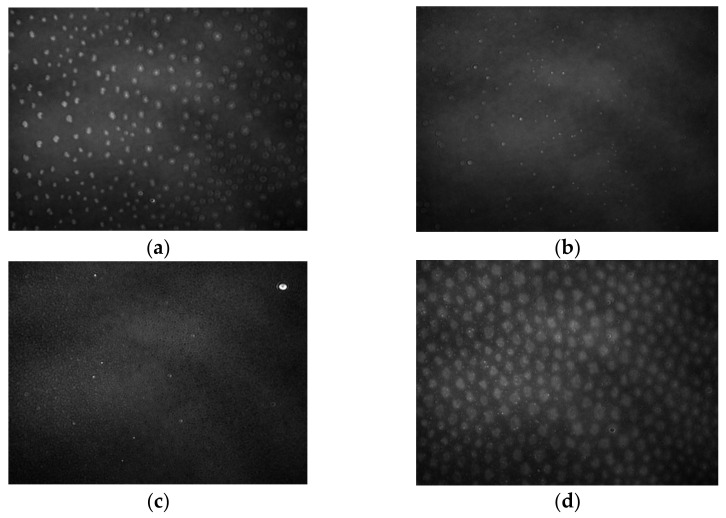
BAM images taken for the PC–K system in a 1:1 ratio at four surface pressure: (**a**) 0.11 mN/m, (**b**) 7.83 mN/m, (**c**) 24.71 mN/m, (**d**) 48.16 mN/m.The images were captured during compression at constant temperature and surface pressure in the field of view 3.6 × 4.0 mm. A black glass plate immersed in the subphase absorbed the refracted beam. The resolution of image was approx. 6 μm/pixel.

**Figure 12:**
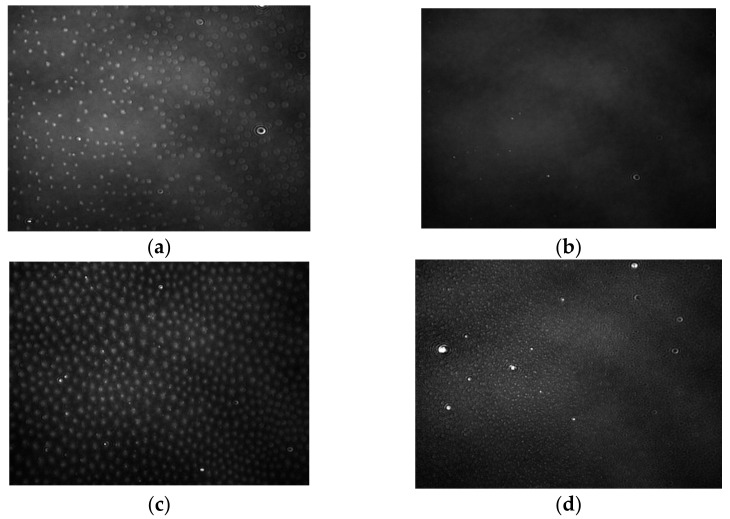
BAM images taken for the PC–M system in a 1:1 ratio at four surface pressure: (**a**) 0.02 mN/m, (**b**) 9.44 mN/m, (**c**) 28.31 mN/m, (**d**) 54.42 mN/m.The images were captured during compression at constant temperature and surface pressure in the field of view 3.6 × 4.0 mm. A black glass plate immersed in the subphase absorbed the refracted beam. The resolution of image was approx. 6 μm/pixel.

In Figure 11 and Figure 12, BAM images recorded for the PC–K and PC–M complex systems are presented in a 1: 1 ratio. Descriptions of the individual phase states are presented in Section 2.2. Initially, for both complexes, we observed the existence of a gas phase (Figure 11a and Figure 12a), where the molecules were located at long distances. A system creates this phase with good miscibility of the agglomerates. As the film compressed, there was a transition to the stretched liquid phase (Figure 11b and Figure 12b) that enters into the condensed liquid phase (Figure 11c and Figure 12c). The interaction between the molecules then increases—the hydroxyl groups develop strong interactions through hydrogen bonds. In hydrocarbon chains, the hydrophobic effect and van der Waals interactions are of the greatest importance. The creation of domains is observed. The monolayer particles are packed to the maximum and form a rigid layer with the properties of a crystalline state. The interactions among the components suggest the formation of a complex responsible for the maximum stability of the system. Further compression causes blurring of the image, which proves that the monolayer collapses under the surface of the subphase. The three-dimensional phase structures characterized by high light reflectance were visible during the refraction of the monolayer; they took the form of bright spots (Figure 11d and Figure 12d). This was due to the mechanical instability of the monolayer.

### 2.3. Microelectrophoretic Experiments

The electrical property parameters of lipid membranes, such as surface charge density and zeta potential, are important values that describe many natural cells’ processes. Many biological membranes possess surface charges due to the presence of biological membrane lipids and proteins. They contain groups that are charged at the appropriate pH. One method of determining membrane surface charge is to measure the electrophoretic mobility of cells [32]. This approach provides an estimate of the average charge density over the entire cell membrane. Changes in the surface charge density of a living cell can commonly occur, including the accompaniment of diseases in cells, since membrane surface charge is influenced by many different factors, such as membrane composition. This contributes significantly to the understanding of many disease mechanisms in many studies of natural membranes, and they are artificial models that provide valuable information about in vivo processes [33,34].

**Figure 13:**
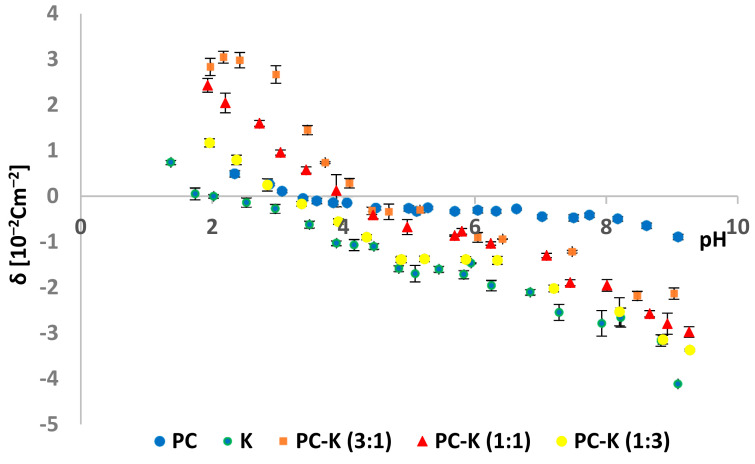
Dependence of PC and PC–K membrane surface charge densities vs. the pH of the electrolyte solution.

**Figure 14:**
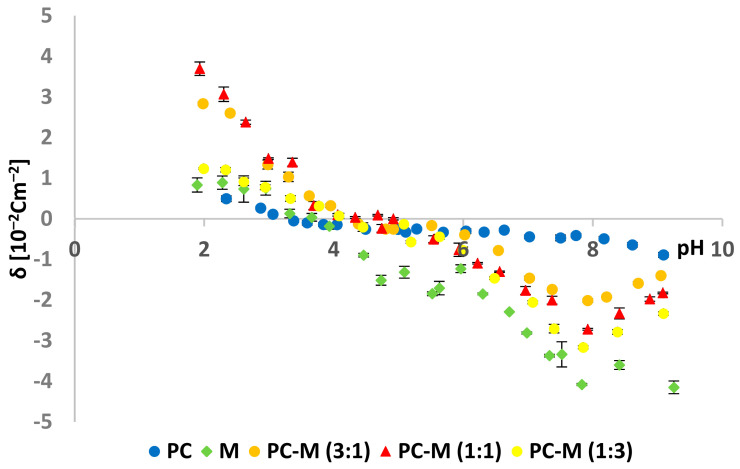
Dependence of the PC and PC–M membrane surface charge densities vs. the pH of the electrolyte solution.

The effect of kaempferol and myricetin on the surface charge density of PC membranes was studied. The surface charge densities of PC–K and PC–M mixture were shown vs. pH (2–10 range) and the molar composition of the liposome components in the membranes (Figure 13 and Figure 14, respectively).

The dependence of the surface charge density on the pH of the applied electrolyte for phosphatidylcholine, kaempferol, and their mixture in the ratios of 1:3, 1:1, and 3:1 was determined (Figure 13). It was observed that with the increase in the kaempferol content in the mixtures, the isoelectric point moved close to the isoelectric point of pure PC values. There was also an increase in the surface charge density both at pH ~ 2 and pH ~ 9.

The phosphatidylcholine–myricetin system (Figure 14) was examined analogously. It was observed that, as the content of myricetin increased in the tested mixtures, the isoelectric point moved towards higher pH values. In the phosphatidylcholine–myricetin system, in all examined ratios, we observed an increase in the positive surface charge density at pH ~ 2 and an increase in the negative charge at pH ~ 9.

Table 2 and Table 3, present the results summarizing the effect of the composition of liposomes formed from the tested components on the isoelectric points and the value of the surface charge densities for the PC–K and PC–M systems, respectively. We express the summarized results as the mean value with the designated standard deviation. We performed the analysis using standard statistical analysis.

Modifying PC membranes with kaempferol and myricetin caused changes in the surface charge density (Table 2 and Table 3, Figure 13 and Figure 14) The liposome’s surface charge depended on the electrolyte ions and electrolyte concentration used in measurements, pH, and membrane composition. We hope that the description of the selected flavonoids’ interactions with natural membranes may contribute to their use as therapeutically active substances. The determined data show that the modification of PC membranes with kaempferol and myricetin caused changes in the analyzed parameters, characterizing model lipid membranes.

Comparing the two tested systems, PC–K and PC–M, the following conclusions can be made:

- The isoelectric point for the PC lipid with kaempferol (e.g., for a 1:1 ratio) lies at lower pH values (~4.0) compared to the PC–M system (pH ~ 4.4);
- The surface charge density values for the PC–K system in the range of pH 2–9 showed values from 3.0 to −4.1 × 10^−2^ C m^−2^, while for the PC–M system, these values were higher and were 3.7 × 10^−2^ C m^−2^ (for PC–M (ratio 1:1) at pH ~ 2) to −4.2 × 10^−2^ C m^−2^ (for pure M).

Myricetin and kaempferol are flavonoids present in many foods that have shown biological activities in numerous studies and have potential use as nutraceuticals [35,36]. The chemical stability of myricetin depends on pH and temperature [36]. Depending on environmental conditions, myricetin may exert both strong antioxidant and pro-oxidative effects in vitro. Buchter et al. [37] attributed a direct antioxidant effect to it. On the other hand, Chobot and Hadacek [38] demonstrated the pro-oxidative properties of myricetin in reducing molecular oxygen to reactive oxygen species (ROS). Myricetin has shown antioxidant properties and free radical scavenging action [39]. These activities seem to support a wide range of positive outcomes, including antiplatelet, antihypertensive, immunomodulating, anti-inflammatory, antiallergic, analgesic, and antitumor [36,40,41,42,43,44,45]. Kaempferol is a natural flavonol-type flavonoid and has hydrophobic properties. The most well-known properties of kaempferol are its anti-inflammatory effects. Kaempferol has been demonstrated to have beneficial effects on chronic inflammatory diseases. The second most important feature of kaempferol is in the prevention of cancer. Its anticancer role has been demonstrated in many types of cancer (e.g., breast cancer, cervical cancer, hepatocellular carcinoma, ovarian cancer, gastric cancer, lung cancer) [46].

In conclusion, our data demonstrated that myricetin and kaempferol affect the physicochemical properties of the tested model structures (i.e., monolayers and liposomes). A thorough understanding of the physicochemical and electrical properties of cell membranes is, in turn, necessary to continue the study of the mechanisms of substance transport or the transmission of information through biological membranes. Before any substance can be approved for widespread use as a medicine, it undergoes a number of tests and studies. All of this is conducted to ensure that it is completely safe for human life and health. Therefore, the initial conduct of basic research on cellular models is most expedient from the point of view of protecting the body during therapy. Initial studies on models are reasonable, because even if the drug has a destructive effect on the artificial membrane, this effect can be prevented and research continued on already living cells.

## 3. Materials and Methods

### 3.1. Materials

*Membrane-forming materials*: Kaempferol (≥98%) and myricetin (97–103%) were supplied by POL-AURA, Dywity, Poland, and used without further purification. The source of lipids was the DPPC (≥99%) from SIGMA–ALDRICH (St. Louis, MO, USA). The molecular weights of the kaempferol, myricetin, and DPPC were approximately 286.24, 318.24, and 734.04 g mol^−1^, respectively.

*Electrolyte solutions:* The electrolyte solutions for the monolayer (pure water) and microelectrophoresis (0.155 M NaCl) were prepared using ultrapure water (obtained by the Milli-Q plus water purification system (Millipore, Burlington, MA, USA) with a resistivity of 18.2 MΩ cm).

#### 3.1.1. Monolayer Preparation—Spreading Solvent and Subphase

The chloroform (≥98.5%) was supplied by Avantor Performance Materials Poland S.A. (Gliwice, Poland) and was used as a spreading solvent for the used substances. The concentration of the tested solutions was 1 mg cm^−3^. To prepare a kaempferol or myricetin solution, initially, the substance was dissolved in a small amount of ethanol (≥99.8%, Avantor Performance Materials Poland S.A., Gliwice, Poland), and then chloroform was added. The ultrapure water subphase solution was used as the subphase. High-purity methanol (≥99.8%, Avantor Performance Materials Poland S.A., Gliwice, Poland) was used to clean the Langmuir trough.

#### 3.1.2. Liposomes Preparation

*Liposome-forming solutions*: The solutions for liposome formation were composed of 10 mg·cm^−3^ of the substances (PC, K, M) in chloroform (anhydrous, ≥98.5%, Avantor Performance Materials Poland S.A., Gliwice, Poland). The components were mixed in three molar ratios (3:1; 1:1; 1:3). Then, the chloroform was evaporated under a stream of argon to obtain a dry residue. The resulting residue was hydrated with an electrolyte solution (0.155 M NaCl).

*Preparation of liposomes:* Liposomes were prepared by sonicating the suspension. To liposomes formation, solutions (concentration 10 mg cm^−3^) of phosphatidylcholine, kaempferol, and myricetin, and their mixtures in a weight ratio were used argon to evaporate the solvent and protect the resulting layer from oxidation. Next, we added 5 mL 0.9% NaCl and, using an ice bath, sonication was conducted (5 times by 1.5 min) with a UD-20 ultrasonic disintegrator (Techpan, Pulawy, Poland).

### 3.2. Monolayer Measurements

*Working conditions and experimental procedure:* The surface tension was measured at the air/water interface (22 °C) using a KSV NIMA BAM apparatus by Biolin Scientific (Helsinki, Finland). The monolayers were prepared by using a microsyringe to spread a calculated volume of the substance in chloroform on the aqueous subphase. A total of 15 min was allowed to evaporate off the spreading solvent and monolayer equilibration before starting the measurement. Then, the monolayer was subjected to continuous compression with a barrier to obtain π–A isotherms. These measurements were carried out using the Langmuir method.

The KSV NIMA BAM apparatus consisted of a large-type Langmuir tub (KN 1006) combined with a Brewster angle microscope (ultraBAM nanofilm from Accurion, Goettingen-Germany)—KSV NIMA Microscopy Trough. The tub tray with an area of 841 cm^2^ (external dimensions of the tray 580 × 145 × 4) was made of pure Teflon (PTFE (polytetrafluoroethylene)), which, as a hydrophobic material, prevents the contents from spilling over the edges of the tray. The total volume of the subphase that can be accommodated by the tray was 336 cm^3^. The measurement set also included two symmetrical polyoxymethylene barriers under the trade name Delrin. The barriers were reinforced with metal frames that prevented the deformation of the Teflon part after prolonged use. An important element of the Langmuir bathtub is a very sensitive surface pressure sensor—Wilhelmy’s platinum plate, which can be replaced by a paper sensor. Changes in surface pressure, temperature, and the position of the barriers were monitored by a special detection unit.

Before the experiment was started, the Teflon trough was carefully cleaned and rinsed with methanol and ultrapure water. The experimental system was enclosed in an acrylic box to minimize water evaporation, ensure high humidity, and avoid contamination. The reported values were highly reproducible and represented the average of at least five experiments. Standard deviations for the measurements were less than 1%.

**Figure 15:**
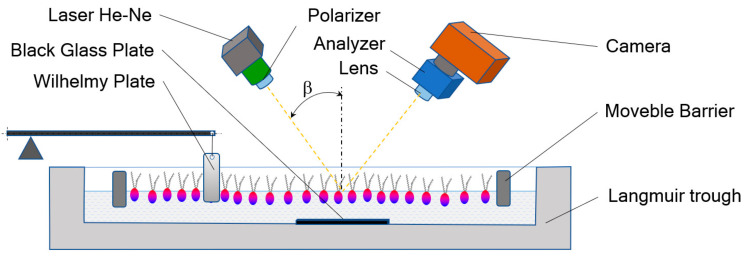
Schematic of the experimental setup of Brewster angle microscopy.

The basic research method used in these studies was the Langmuir method with Brewster angle microscopy. The test device consisted of a Teflon trough and two symmetrical rails. The receptor part was a platinum Wilhelmy plate connected to a sensitive electromagnet. The camera consisted of a laser, polarizer, lens, analyzer, and CCD camera (Figure 15).

The BAM technique was used in the study of monolayers based on sources for water-soluble amphiphilic substances. This method is based on the change in the refractive index at the water/air interface as a result of the Langmuir monolayer formation on it. At the border of two phases, differing in density, the incident beam of radiation is partially reflected from the interface, and the remaining part goes to the adjacent phase. If we are dealing with an interface where the refractive indexes differ and the incident light beam is polarized, then the light is not reflected. Such a phenomenon is characteristic only for a specific angle, called the Brewster angle (*β_B_*), which we can define as:

(12) $$\tan\beta_{B}=\frac{n_{1}}{n_{2}}$$

This angle at the water–air interface takes the value, and the generated surface image is black. By adding a substance that will form a thin layer, the refractive index will change. The formed monolayers have a specific thickness and refractive index, as a result of which part of the polarized light is reflected and will be recorded by a sensitive detector. The reflected light beams will show a different degree of intensity, as the content of particles and their packing density differ. The obtained BAM images, in conjunction with the analysis of π-A isotherms, allow the characterization of changes in the morphology of the monolayer and the formation of domains or aggregates during phase transitions accompanying the process of monolayer compression [47,48,49].

### 3.3. Microelectrophoretic Measurements

The essence of using the microelectrophoresis method is to obtain a graph of the surface charge density dependence on the pH value. The use of Zetasizer Nano ZS (Malvern Instruments, Malvern, UK) apparatus allows one to obtain data on the microelectrophoretic mobility of the studied systems using the laser Doppler microelectrophoresis (LDE) technique. All measurements were performed as a function of pH using a WTW InoLab pH 720 laboratory meter (WTW, Weinheim, Germany). Liposomes suspended in an electrolyte solution (0.155 mol dm^−3^ NaCl) were titrated to the given pH value (range 2–10, every ± 0.3 units) with sodium hydroxide or hydrochloric acid. Six measurements were made (each covering 100–200 series, duration 5 s) for each pH value for each sample. The experiments were carried out three times [23,24,32,33,34]. The value of electrophoretic mobility enables the determination of the surface charge density according to the equation [50]:

(13) $$\delta =\frac{\eta \cdot u}{d}$$

where u is the electrophoretic mobility, η is the viscosity of the solution, and d is the thickness of the diffuse layer.

Equation (14) allows one to determine the thickness of the diffusion layer [51]:

(14) $$d=\sqrt{\frac{\varepsilon \varepsilon_{0}RT}{2F^{2}I}}$$

where *εε*_0_ is the permeability of the electric medium, *R* is the gas constant, *T* is the temperature, *F* is the Faraday number, and I is the ionic strength of 0.9% NaCl.

## 4. Conclusions

In the above study, the influence of the biologically active compounds kaempferol and myricetin on the properties of model biological membranes was investigated. The complex formation between the components of the mixture of phosphatidylcholine and the tested compounds was assumed. A series of analyses were carried out to determine the specific surface area, constant stability of the complex, and its formation energy. The obtained values of the specific surface area of the complex were consistent with the theoretical values. The high values of the stability constants and the negative energy of complex formation confirm the correctness of the assumption that phosphatidylcholine, together with kaempferol and myricetin, forms complexes in the ratio of 1:1. Based on the data presented in the article, it can be concluded that the tested compounds affected the properties of model cell membranes made of phosphatidylcholine. Mathematically computed and experimentally validated data are important for understanding various phenomena occurring in natural membranes.

## Figures and Tables

**Table 1:** Calculated physicochemical parameters for examined complexes: phosphatidylcholine–kaempferol (PC–K) and phosphatidylcholine–myricetin (PC–M).

System	Surface Area of Complex(Å^2^ Molecule^−1^)	Stability Constant (m^2^∙mol^−1^)	Complex Formation Energy (kJ mol^−1^)
PC–K	64	2.8 × 10^6^	−37 ± 1
PC–M	60	2.1 × 10^6^	−36 ± 1

**Table 2:** The surface charge densities and isoelectric points for the PC–K system.

Examined System	Isoelectric Point	Surface Charge Density δ (10^–2^ C m^–2^)	
		Low pH Values	High pH Values
PC	~3.2	0.8 ± 0.1	−0.9 ± 0.1
PC–K (3:1)	~4.1	2.8 ± 0.2	−2.1 ± 0.3
PC–K (1:1)	~4.0	2.4 ± 0.2	−3.0 ± 0.3
PC–K (1:3)	~3.4	1.1 ± 0.1	−3.4 ± 0.5
K	~2.0	0.1 ± 0.0	−4.1 ± 0.6

**Table 3:** The surface charge densities and isoelectric points for the PC–M system.

Examined System	Isoelectric Point	Surface Charge Density δ (10^−2^ C m^−2^)	
		Low pH Values	High pH Values
PC	~3.2	0.8 ± 0.1	−0.9 ± 0.1
PC–M (3:1)	~4.1	2.8 ± 0.6	−1.4 ± 0.2
PC–M (1:1)	~4.3	3.7 ± 0.4	−1.8 ± 0.2
PC–M (1:3)	~4.1	1.2 ± 0.2	−2.3 ± 0.2
M	~3.7	0.9 ± 0.2	−4.2 ± 0.4

## Data Availability

The data presented in this study are available on request from the corresponding author.
